# Supplementary material for: Gut butyrate-producers confer post-infarction cardiac protection
Source: Nat Commun. 2023 Nov 9;14:7249. doi: 10.1038/s41467-023-43167-5 (PMC10636175; doi:10.1038/s41467-023-43167-5)
Supplement: Supplementary file 1 — Supplementary information [file 41467_2023_43167_MOESM1_ESM.pdf]

Supplemental Information for

Gut butyrate-producers confer post-infarction cardiac protection

Hung-Chih Chen *et al.*

Corresponding author: Patrick C.H. Hsieh, phsieh@ibms.sinica.edu.tw

This PDF file includes:

Supplemental Tables 1 to 6

Supplemental Figures 1 to 12

Characteristic	No. (%) Total (n = 147)	Control (n = 70)	STEMI (n = 77)	P Value
Age, mean (SE), y	54.0 (1.0)	52.0 (1.4)	55.7 (1.2)	0.052
Male	115 (78.2)	43 (61.4)	72 (93.5)	<0.0001
**Location**				
Northern Taiwan	26 (17.7)	7 (10.0)	19 (24.7)	
Middle Taiwan	48 (32.6)	42 (60.0)	6 (7.8)	
Southern Taiwan	73 (49.7)	21 (30.0)	42 (54.5)	
**Comorbidities and risk factors**				
Hypertension	46 (31.3)	24(34.3)	23 (29.9)	0.57
Hyperlipidemia	77 (52.4)	25 (35.7)	52 (67.5)	<0.0001
Diabetes	29 (19.7)	14 (20.0)	15 (19.5)	0.94
**Presenting characteristics**				
**Body mass index**				
Underweight(<18.5)	1 (0.7)	1 (1.4)	0 (0.0)	
Normal (18.5 to <25)	48 (32.7)	20 (28.6)	28 (36.4)	0.35
Overweight (25 to 30)	60 (40.8)	23 (32.8)	37 (48.1)	0.15
Obese (≧30)	17 (11.6)	4 (5.7)	13 (16.9)	0.63

Supplementary Table 1. Clinical summary of recruited participants.

The data were analyzed with two-sided unpaired Student's *t*-test.

Bacteria		Sequence (5'----3')
Anaerotruncus colihominis DSM 17241	(F)	CTAAAACAGAGGGCGGCGAC
	(R)	CTTCGGGTGTTACCCGGACTC
Clostridium symbiosum ATCC 14940	(F)	AACTGGAGTGTCGGAGAGGT
	(R)	TTCATCGTTTACGGCGTGGA
Coprococcus comes ATCC 27758	(F)	GGCGTGTAATGACGCCTTTT
	(R)	AGTCTCTCCAGAGTGCCCAT
Ruminococcus torques ATCC 27756	(F)	CGAGGTGGAGCAAATCCCAA
	(R)	ACTGACTTCGGGCGTTACTG
Bacteroides caccae ATCC 43185	(F)	ATGGGGAAACCCATACGCC
	(R)	CCAGAGTCCTCAGCATGACC
Bacteroides thetaiotaomicron VPI-5482	(F)	GGGCAGTGATCTACGTGTCAAG
	(R)	CTGCATCGTACCCAAAATCGTCTG
Providencia stuartii ATCC 29914	(F)	TCCCTAGAGGAGTGGCTTCC
	(R)	CTCCCGAAGGCACTAAAGCA
Collinsella aerofaciens ATCC 25986	(F)	CTCTCCGGAGGGAAGCGAG
	(R)	TGTCTCAGTCCCAATCTGGC
Bifidobacterium adolescentis ATCC 15703	(F)	CCGGTGTAACGGTGGAATGT
	(R)	GACACGGAGACCGTGGAATG
Bifidobacterium ruminantium	(F)	TCCTATCAGGTAGTCGGCGG
	(R)	GCTTGCTCCCAGTCAAAAGC
Streptococcus parasanguinis ATCC15912	(F)	ATGGGGTGACCATCGCAAAA
	(R)	GAGTCAAAACCGTTGCGGTC
Streptococcus salivarius	(F)	GTTATGAGCTCAGGCTCGCT
	(R)	GCAGCAATTCCGCCTTCTTT
Butyricimonas virosa JCM 15149	(F)	AAGGATGACGAGTCATTCGATGC
	(R)	CTTCACTTGTTCCGCCTCCC
Subdoligranulum variabile	(F)	GATCCGGCATCGGATTGAGG
	(R)	GTGCAATATCCCCCACTGCT

Supplementary Table 2. Primer sets for bacterial validation.

Mean

	1557 taxa (all taxa)						1366 taxa (genus-only)					
	ratio	raw	z-score	min-max	maxabs	robust	ratio	raw	z-score	min-max	maxabs	robust
training	1.000	1.000	1.000	1.000	1.000	1.000	1.000	1.000	1.000	1.000	1.000	0.998
validation	0.873	0.892	0.895	0.902	0.899	0.897	0.877	0.902	0.900	0.899	0.893	0.888
test	0.851	0.863	0.856	0.850	0.857	0.882	0.864	0.884	0.848	0.847	0.831	0.862

Standard deviation

	1557 taxa						1366 taxa					
	ratio	raw	z-score	min-max	maxabs	robust	ratio	raw	z-score	min-max	maxabs	robust
training	0.000	0.000	0.000	0.000	0.000	0.000	0.000	0.000	0.000	0.000	0.000	0.006
validation	0.045	0.038	0.036	0.024	0.031	0.025	0.041	0.031	0.031	0.041	0.033	0.023
test	0.055	0.043	0.067	0.064	0.056	0.038	0.062	0.023	0.083	0.047	0.074	0.049

Supplementary Table 3. Mean and standard deviation values of training, validation, and test with six major metrics used for machine learning.

	**Accuracy**	**AUC**	**Recall**	**Precision**
w/ calibration	0.78 (0.046)	0.88 (0.051)	0.83 (0.076)	0.76 (0.083)
w/o calibration	0.80 (0.027)	0.88 (0.023)	0.82 (0.068)	0.79 (0.066)
p-value of *t*-test	0.21	0.95	0.72	0.30

Supplementary Table 4. Mean (standard deviation in parentheses) values of four major metrics and their *t*-test results

Paper	Trait	Samples	Cases	Controls	Taxa	Level	Method	Accuracy	AUC	Recall	Precision
Current study	STEMI	144	70	70	1557 ASV	Genus	Mixed	0.78	0.88	0.83	0.76
Aryal et al., 2020	CVDs	951	478	473	25-500 OTU	Genus	RF	n/a	0.70	0.69	n/a
Pasolli et al., 2016	Liver cirrhosis	232	118	114	542	Species	SVM	0.83	0.92	0.83	0.84
	Colorectal Cancer	121	48	73	503	Species	RF	0.80	0.87	0.81	0.82
	Obesity	253	164	89	465	Species	RF	0.64	0.66	0.64	0.54
	IBD	110	25	85	443	Species	RF	0.81	0.89	0.81	0.72
	Diabetes	96	53	43	381	Species	RF	0.70	0.76	0.70	0.73

Supplementary Table 5. Comparison of prediction accuracy with published results.

STEMI, ST-elevation myocardial infarction; CVD, cardiovascular disease; IBD, inflammatory bowel disease; ASV, amplicon sequence variant; OTU, operational taxomonic unit; RF, random forest; SVM, support vector machine.

	Precursor ion Mass (m/z)	Product ion Mass (m/z)	DP (V)	CE (eV)
BHB	103.1	59.1	-50	-10
BHB-d4	107.1	59.1	-50	-13
C_{Sp1}	103.1	59.0	-50	-10
C_{Sp2}	103.1	59.0	-50	-10
C_{Sp3}	103.1	59.0	-50	-10
C_{Bv1}	103.1	59.0	-50	-10
C_{Bv2}	103.1	59.0	-50	-10
C_{Bv3}	103.1	59.0	-50	-10
C_{Ba1}	103.1	59.0	-50	-10
C_{Ba2}	103.1	59.0	-50	-10
C_{Ba3}	103.1	59.0	-50	-10

Supplementary Table 6. Ion-pair selection for liquid chromatography-tandem mass spectrometry (LC-MS/MS) with multiple reaction monitoring (MRM) mode. BHB, β-hydroxybutyrate; BHB-d4, Sodium D-β-Hydroxybutyrate-d4 as an internal standard; DP: declustering potential; CE, Collision Energy; C_{Sp}, conditioned medium of *Streptococcus parasanguinis*; C_{Bv}, conditioned medium of *Butiricimonas virosa*; C_{Ba}, conditioned medium of *Bifidobacterium adlescentis*.

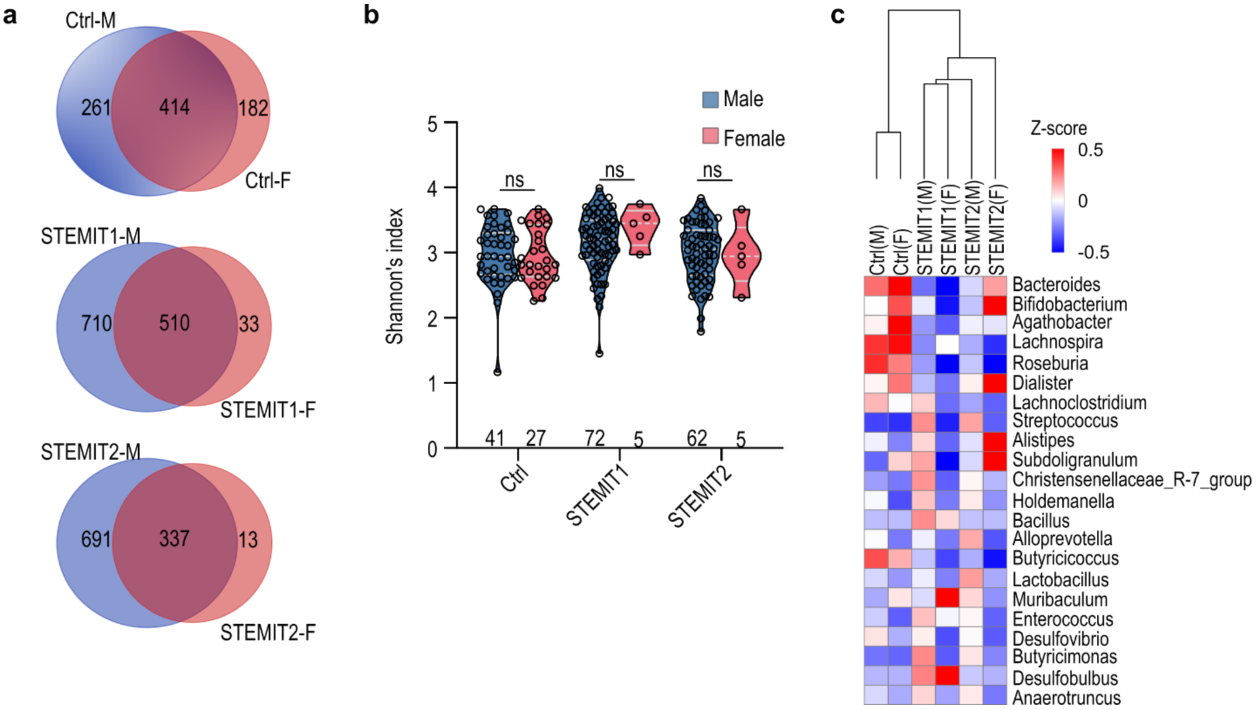

Supplementary Figure 1. The gender effects on gut microbiota.

(**a**) Venn diagram showing overlapping amplicon sequence variants (ASVs) between male (Ctrl-M, STEMIT1-M and STEMIT2-M) and female (Ctrl-F, STEMIT1-F and STEMIT2-F) samples. (**b**) Shannon's index of Ctrl and STMEI gut microbiota in male and female samples. (**c**) Differentially abundant bacterial genera in Ctrl and STEMI samples of different genders. The number of biologically independent samples are indicated in each chart. Data in (**b**) were analyzed with two-way ANOVA and are represented as mean ± SEM.

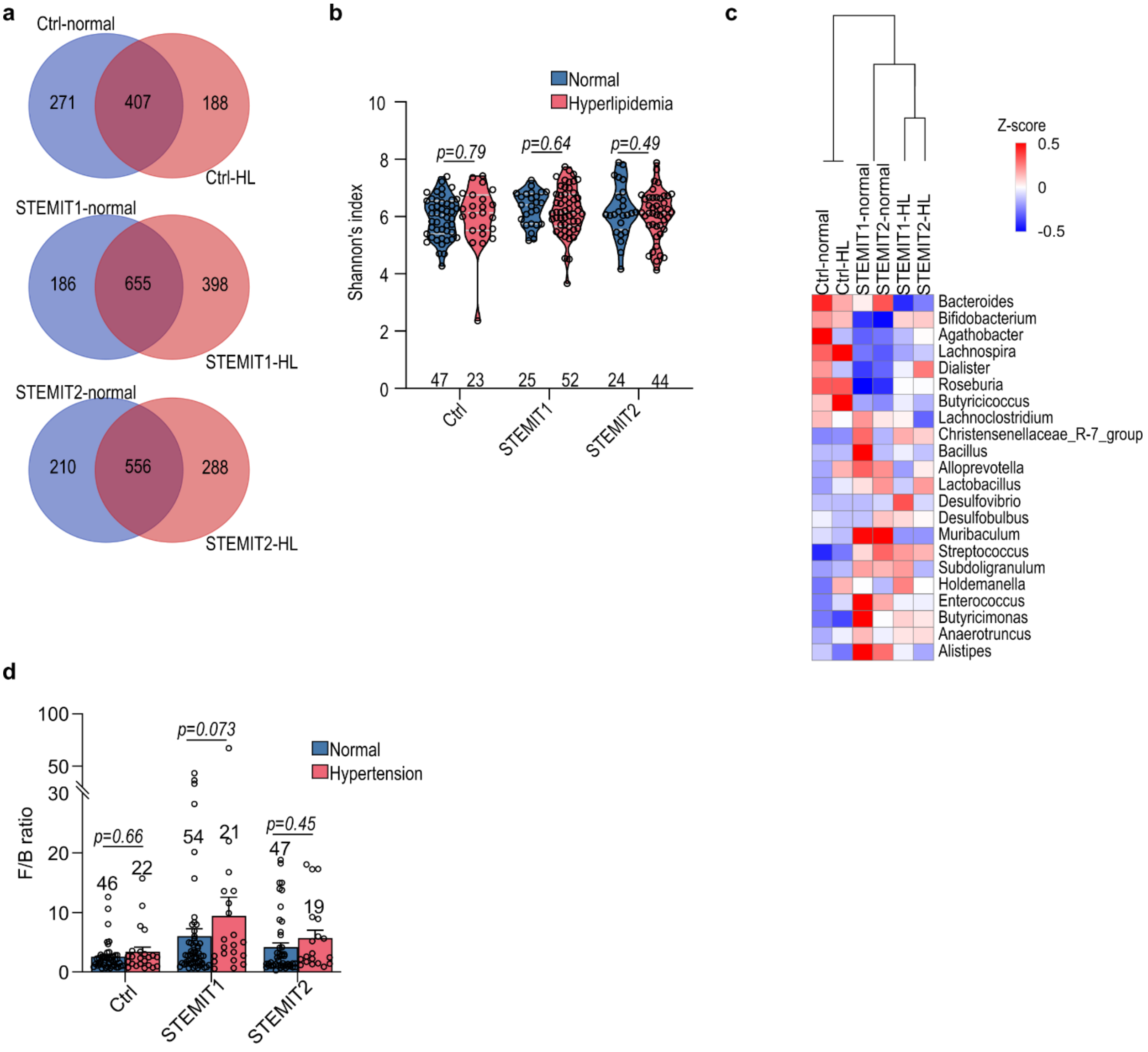

Supplementary Figure 2. The effects of hyperlipidemia and hypertension on gut microbiota. (**a**) Venn diagram showing overlapping ASVs between normal (Ctrl-normal, STEMIT1-normal and STEMIT2-normal) and hyperlipidemic (Ctrl-HL, STEMIT1-HL and STEMIT2-HL) samples. (**b**) Shannon's index of Ctrl and STMEI gut microbiota in normal and hyperlipidemic samples. (**c**) Differentially abundant bacterial genera in Ctrl and STEMI samples with/without hyperlipidemia. (**d**) The ratio of Firmicutes/Bacteroidetes (F/B ratio) in human Ctrl, STEMIT1 and STEMIT2 stool samples with/without hypertension. The number of biologically independent samples are indicated in each chart. Data in (**b**, **d)** were analyzed with two-way ANOVA with FDR correction for six groups and are represented as mean ± SEM.

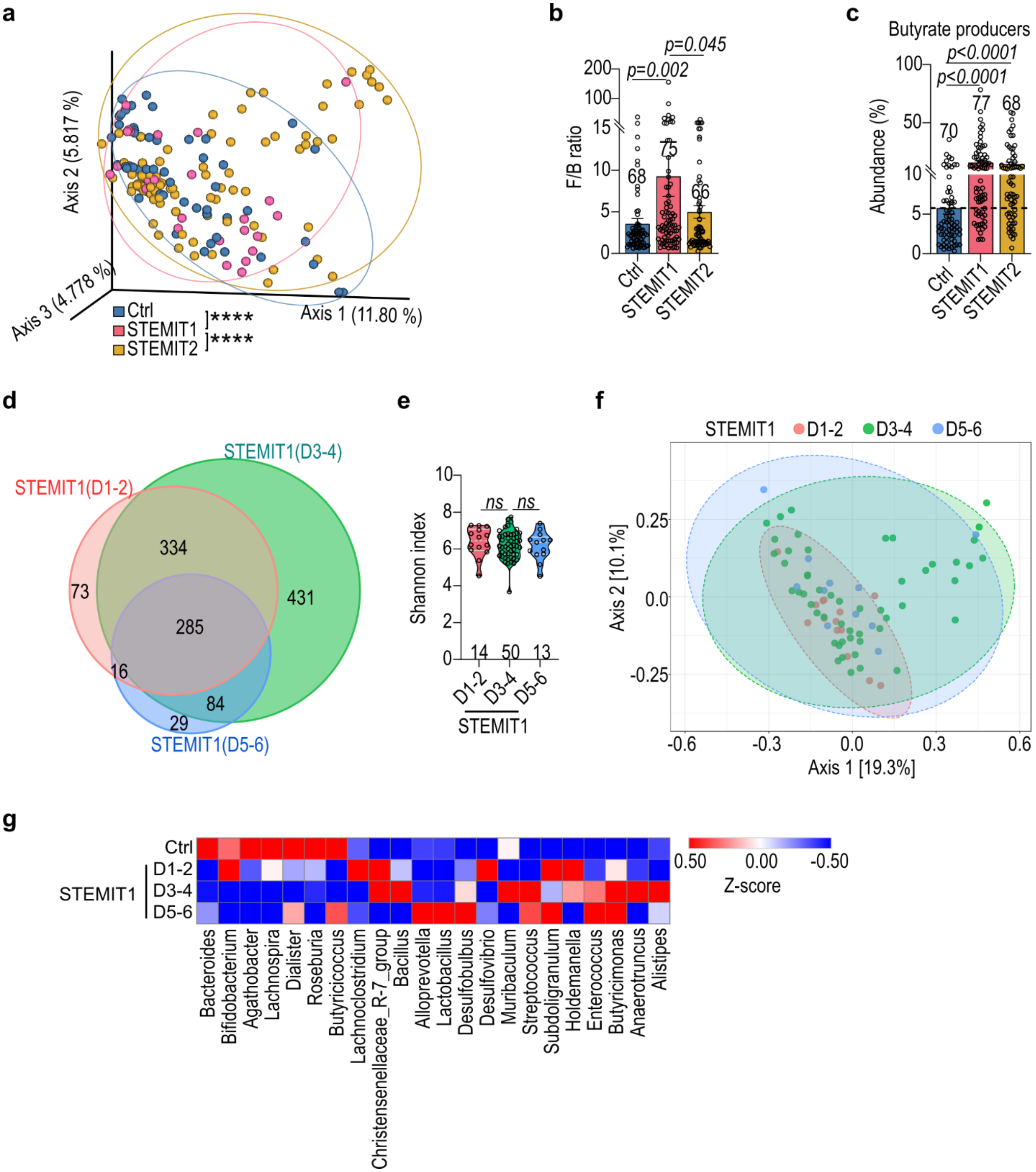

Supplementary Figure 3. Alteration of gut microbiota in human STEMI samples.

(**a**) PCoA of unweighted Unifrac for human Ctrl, STEMIT1 and STEMIT2 gut microbiota. (**b**) The ratio of Firmicutes/Bacteroidetes in human Ctrl, STEMIT1 and STEMIT2 stool samples (vs. STEMIT1). (**c**) Relative abundance of butyrate-producers in human Ctrl, STEMIT1 and STEMIT2

samples (vs. Ctrl). (**d**) Venn diagram showing overlapping ASVs of STEMIT1 samples at day 1-2 (D1-2), day 3-4 (D3-4) and day 5-6 (D5-6). (**e**) The Shannon's index of STEMT1 samples at D1-2, d3-4 and D5-6. (**f**) PCoA of STEMIT1 ASVs at different time points. (**g**) Differential bacterial abundance in STMEIT1 samples at different time points. The number of biologically independent samples are indicated in each chart. Data were analyzed with the Kruskal-Wallis test followed by Dunn's correction. Data are represented as mean ± SEM. ns, not significant; **** p <0.0001.

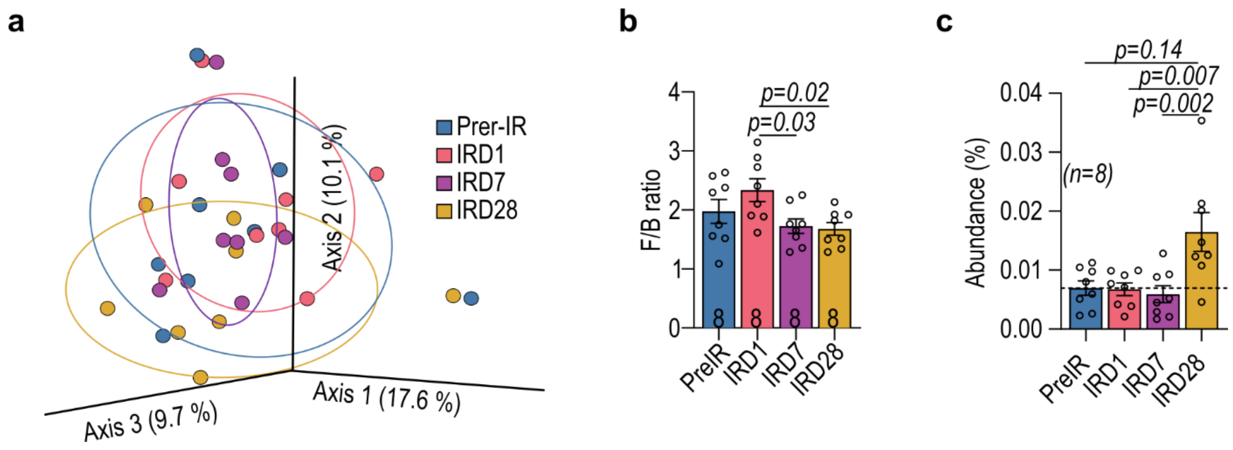

Supplementary Figure 4. Gut microbiota alteration in the nonhuman primate cardiac ischemia/reperfusion (IR) model.

(**a**) PCoA of unweighted Unifrac for nonhuman primate gut microbiota at pre-IR, IRD1, IRD7 and IRD28. (**b**) The ratio of Firmicutes/Bacteroidetes in nonhuman primate cardiac IR model at basal (pre-IR), IRD1, IRD7 and IRD28. (**c**) The abundance of butyrate-producing bacteria. The number of biologically independent animals are indicated in each chart. Data in (**b**, **c**) were analyzed with Kruskal-Wallis test and corrected with FDR. Data are represented as mean ± SEM.

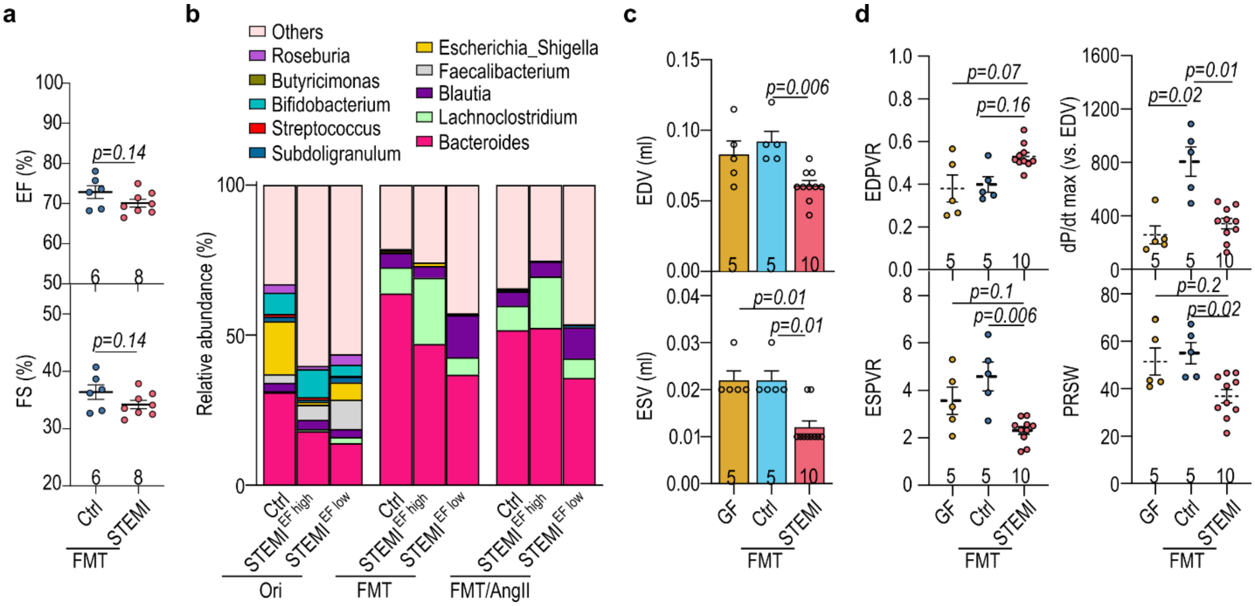

Supplementary Figure 5. The impact of human fecal transplantation on the post-injury cardiac function of germ-free mice.

(**a**) Left ventricular ejection fraction (EF) and fraction shortening (FS) of germ-free (GF) mice receiving control or STEMI fecal microbiome transplantation (FMT) before left anterior descending artery (LAD) ligation. (**b**) Microbiome composition of original human fecal samples (Ori), stool from GF mice with FMT and FMT-GF mice after angiotensin-II challenge (FMT/AngII). (**c**) Changes of left ventricular end-diastolic volume (EDV) and end-systolic volume (ESV) of FMT-GF mice on day 14 after AngII challenge. (**d**) Cardiac catheterization parameters of FMT mice on day 14 after AngII challenge, including end-systolic pressure volume relationship (ESPVR), end-diastolic pressure volume relationship (EDPVR), preload recruitable stroke work (PRSW) and ratio of maximal left ventricular pressure rise (dP/dtmax) to end-diastolic volume (EDV). The number of biologically independent animals are indicated in each chart. Data in (**a**) were analyzed with two-sided Student's *t*-test; data in (**c**, **d**) were analyzed with Kruskal-Wallis test followed by Dunn's correction. Data are represented as mean ± SEM.

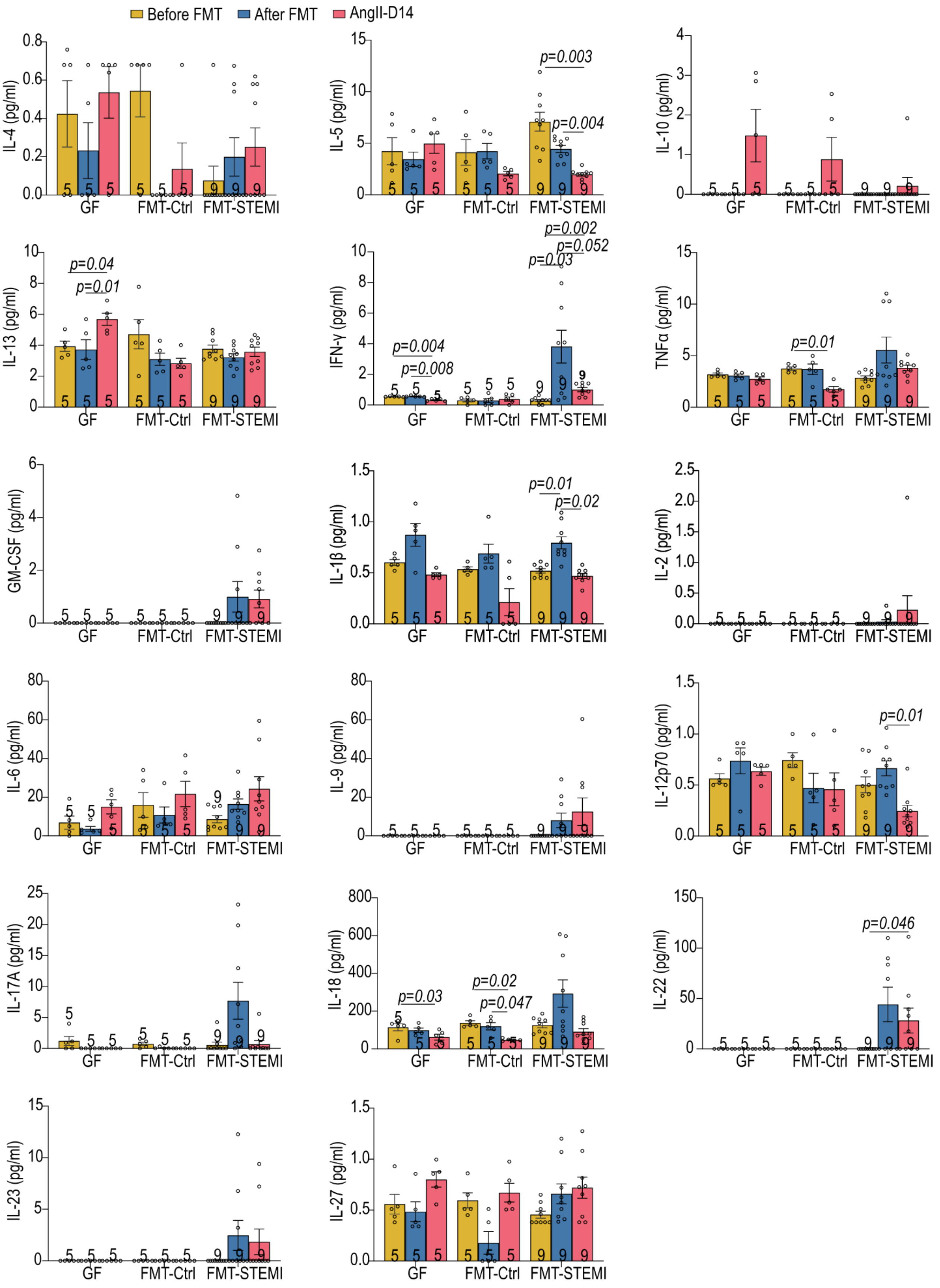
Before FMT
After FMT
AngII-D14
IL-4 (pg/ml)
IL-5 (pg/ml)
p=0.003
p=0.004
IL-10 (pg/ml)
IL-13 (pg/ml)
p=0.04
p=0.01
IFN-γ (pg/ml)
p=0.004
p=0.008
p=0.002
p=0.052
p=0.03
TNFα (pg/ml)
p=0.01
GM-CSF (pg/ml)
IL-1β (pg/ml)
p=0.01
p=0.02
IL-2 (pg/ml)
IL-6 (pg/ml)
IL-9 (pg/ml)
IL-12p70 (pg/ml)
p=0.01
IL-17A (pg/ml)
IL-18 (pg/ml)
p=0.03
p=0.02
p=0.047
IL-22 (pg/ml)
p=0.046
IL-23 (pg/ml)
IL-27 (pg/ml)
GF
FMT-Ctrl
FMT-STEMI

Supplementary Figure 6. The changes of cytokines in GF-FMT mice under Angiotensin-II challenge.

The plasma levels of cytokines in GF-FMT mice were determined using multiplex immunoassays. The number of biologically independent animals are indicated in each chart. Mixed-effects analysis followed by FDR correction was used to analyzed the data. Data are represented as mean ± SEM. IL, interleukin; INF, interferon; TNF, tumor necrosis factor; GM-CSF, granulocyte macrophage-colony stimulating factor.

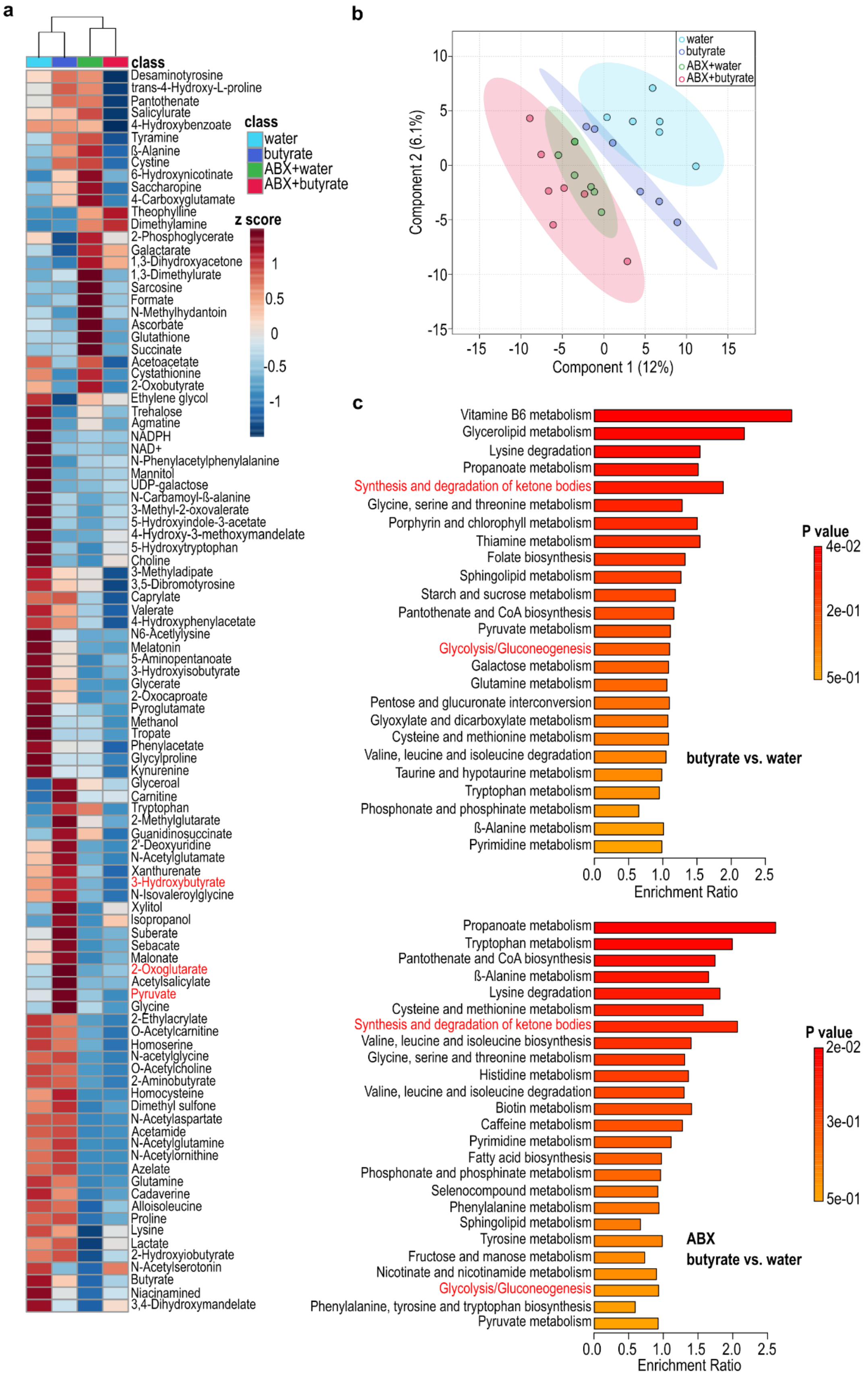
a
class
Desaminotyrosine
trans-4-Hydroxy-L-proline
Pantothenate
Salicylurate
4-Hydroxybenzoate
Tyramine
ß-Alanine
Cystine
6-Hydroxynicotinate
Saccharopine
4-Carboxyglutamate
Theophylline
Dimethylamine
2-Phosphoglycerate
Galactarate
1,3-Dihydroxyacetone
1,3-Dimethylurate
Sarcosine
Formate
N-Methylhydantoin
Ascorbate
Glutathione
Succinate
Acetoacetate
Cystathionine
2-Oxobutyrate
Ethylene glycol
Trehalose
Agmatine
NADPH
NAD+
N-Phenylacetylphenylalanine
Mannitol
UDP-galactose
N-Carbamoyl-ß-alanine
3-Methyl-2-oxovalerate
5-Hydroxyindole-3-acetate
4-Hydroxy-3-methoxymandelate
5-Hydroxytryptophan
Choline
3-Methyladipate
3,5-Dibromotyrosine
Caprylate
Valerate
4-Hydroxyphenylacetate
N6-Acetyllysine
Melatonin
5-Aminopentanoate
3-Hydroxyisobutyrate
Glycerate
2-Oxocaproate
Pyroglutamate
Methanol
Tropate
Phenylacetate
Glycylproline
Kynurenine
Glyceroal
Carnitine
Tryptophan
2-Methylglutarate
Guanidinosuccinate
2'-Deoxyuridine
N-Acetylglutamate
Xanthurenate
3-Hydroxybutyrate
N-Isovaleroylglycine
Xylitol
Isopropanol
Suberate
Sebacate
Malonate
2-Oxoglutarate
Acetylsalicylate
Pyruvate
Glycine
2-Ethylacrylate
O-Acetylcarnitine
Homoserine
N-acetylglycine
O-Acetylcholine
2-Aminobutyrate
Homocysteine
Dimethyl sulfone
N-Acetylaspartate
Acetamide
N-Acetylglutamine
N-Acetylornithine
Azelate
Glutamine
Cadaverine
Alloisoleucine
Proline
Lysine
Lactate
2-Hydroxyiobutyrate
N-Acetylserotonin
Butyrate
Niacinamined
3,4-Dihydroxymandelate
class
water
butyrate
ABX+water
ABX+butyrate
z score
1
0.5
0
-0.5
-1
b
water
butyrate
ABX+water
ABX+butyrate
Component 2 (6.1%)
10
5
0
-5
-10
-15
-15
-10
-5
0
5
10
15
Component 1 (12%)
c
Vitamine B6 metabolism
Glycerolipid metabolism
Lysine degradation
Propanoate metabolism
Synthesis and degradation of ketone bodies
Glycine, serine and threonine metabolism
Porphyrin and chlorophyll metabolism
Thiamine metabolism
Folate biosynthesis
Sphingolipid metabolism
Starch and sucrose metabolism
Pantothenate and CoA biosynthesis
Pyruvate metabolism
Glycolysis/Gluconeogenesis
Galactose metabolism
Glutamine metabolism
Pentose and glucuronate interconversion
Glyoxylate and dicarboxylate metabolism
Cysteine and methionine metabolism
Valine, leucine and isoleucine degradation
Taurine and hypotaurine metabolism
Tryptophan metabolism
Phosphonate and phosphinate metabolism
ß-Alanine metabolism
Pyrimidine metabolism
0.0
0.5
1.0
1.5
2.0
2.5
Enrichment Ratio
P value
4e-02
2e-01
5e-01
butyrate vs. water
Propanoate metabolism
Tryptophan metabolism
Pantothenate and CoA biosynthesis
ß-Alanine metabolism
Lysine degradation
Cysteine and methionine metabolism
Synthesis and degradation of ketone bodies
Valine, leucine and isoleucine biosynthesis
Glycine, serine and threonine metabolism
Histidine metabolism
Valine, leucine and isoleucine degradation
Biotin metabolism
Caffeine metabolism
Pyrimidine metabolism
Fatty acid biosynthesis
Phosphonate and phosphinate metabolism
Selenocompound metabolism
Phenylalanine metabolism
Sphingolipid metabolism
Tyrosine metabolism
Fructose and manose metabolism
Nicotinate and nicotinamide metabolism
Glycolysis/Gluconeogenesis
Phenylalanine, tyrosine and tryptophan biosynthesis
Pyruvate metabolism
0.0
0.5
1.0
1.5
2.0
2.5
Enrichment Ratio
P value
2e-02
3e-01
5e-01
ABX
butyrate vs. water

Supplementary Figure 7. The influence of butyrate on the post-MI plasma metabolic profiling.

(**a**) The heatmap of plasma metabolites of butyrate-supplemented mice. (**b**) The Partial Least Squares Discriminant Analysis (PLS-DA) of plasma metabolites. (**c**) Enrichment of metabolic pathways in butyrate-treated mice without (upper panel) and with antibiotics (lower panel).

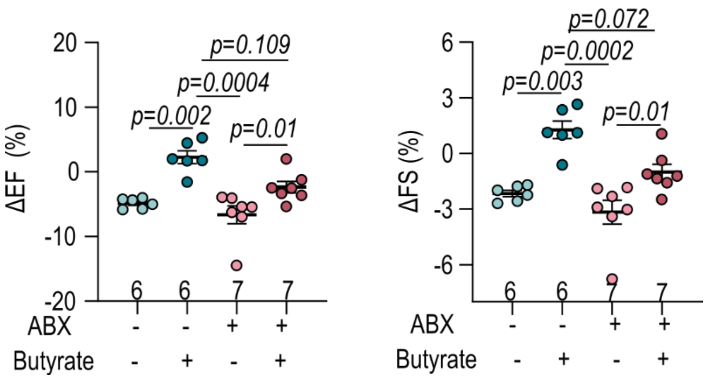

Supplementary Figure 8. The effects of gut microbiota and butyrate on the changes of post-injury cardiac function in SPF mice.

The changes of EF(%) and FS(%) were determined by comparing the pre-MI EF(%) and FS(%) with those on MI day 21. The number of biologically independent animals are indicated in each chart. Data were analyzed with Kruskal-Wallis test followed by FDR correction.

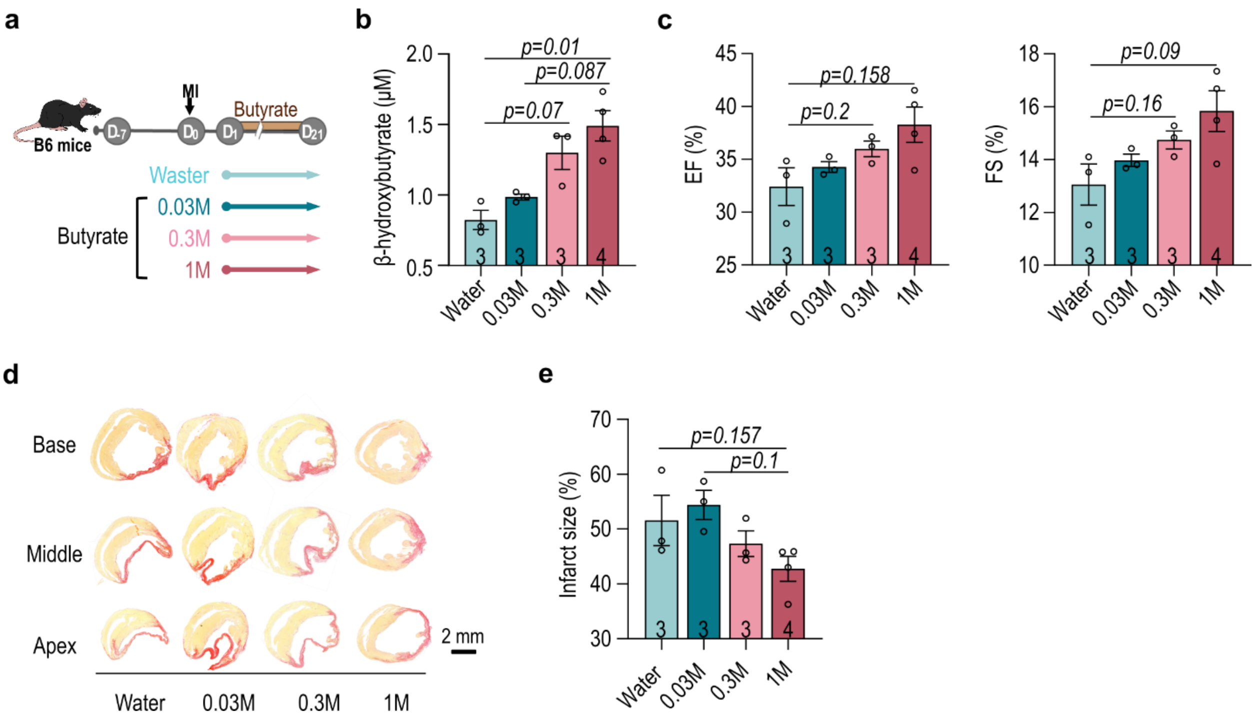

Supplementary Figure 9. The dose-dependent cardioprotection of butyrate in SPF mice.
(**a**) Schematic description of experimental design for butyrate-dose depend effect. (**b**) Plasma level of β-hydroxybutyrate determined with the colorimetric assay. (**c**) Left ventricular EF (%) and FS (%) of mice receiving different doses of butyrate after MI. (**d**) Representative images of cardiac infarct size labeled with picrosirus red and (**e**) statistics. The number of biologically independent animals are indicated in each chart. Data were analyzed with the Kruskal-Wallis test followed by FDR correction. Data are represented as mean ± SEM.

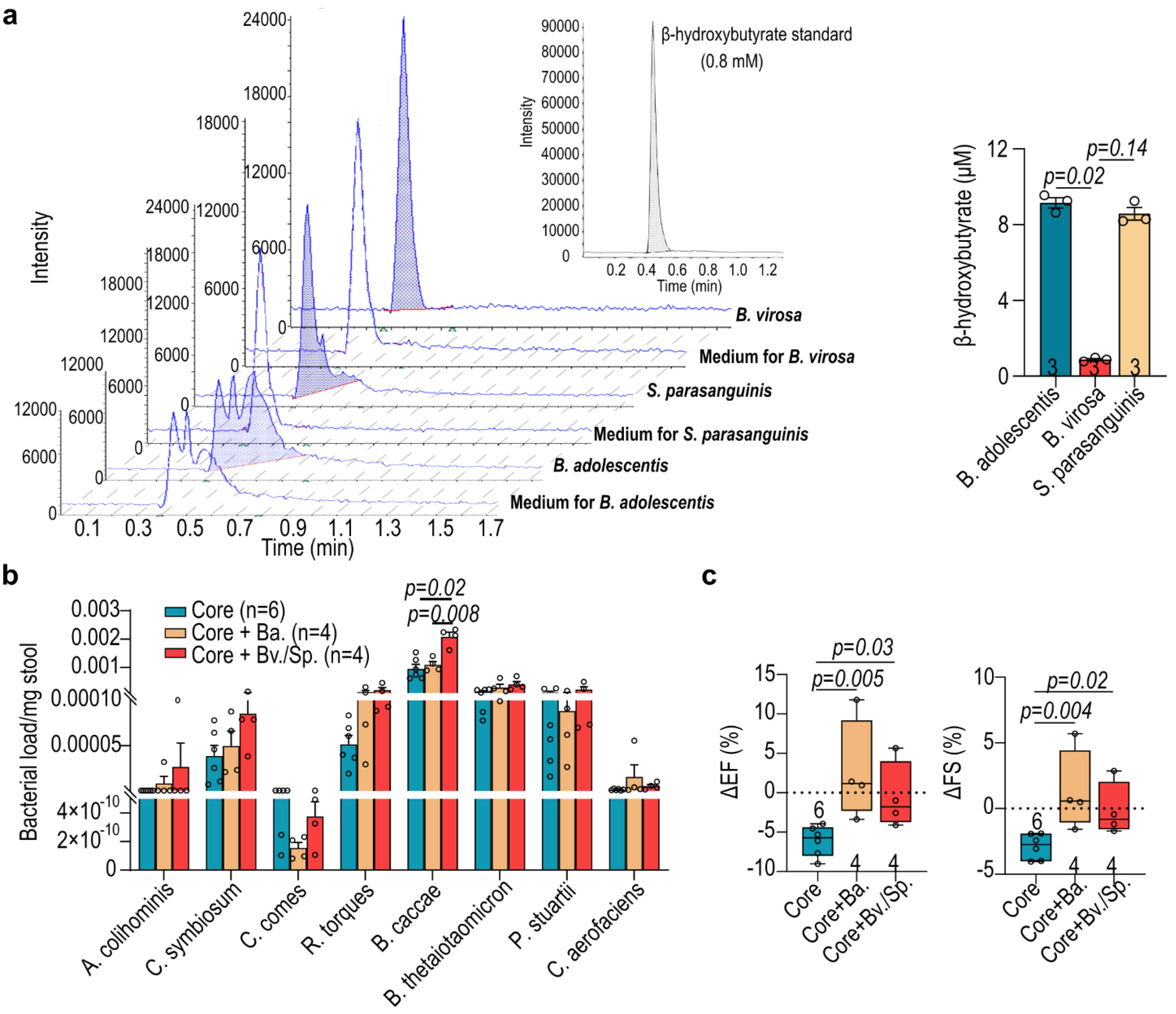

Supplementary Figure 10. Butyrate producers produced β-hydroxybutyarte.

(**a**) In vitro test of β-hydroxybutyarte production by *Bifidobacterium adolescentis* (*B. adolescentis*), *Butyricimonas virosa* (*B. virosa*) and *Streptococcus parasanguinis* (*S. parasanguinis*). The presence of β-hydroxybutyrate in the culture medium was determined with LC-MS normalized with the plain medium for each bacterium. (**b**) Loading of the core bacteria in the gnotobiotic mice was confirmed with qPCR (vs. Core+*Bv./Sp.*). (**c**) Echocardiographic analysis of changes in left ventricular EF (%) and FS (%) in gnotobiotic mice on MI day 21 (vs. Core). The number of biologically independent samples are indicated in each chart. Data were analyzed with the Kruskal-Wallis test followed by Dunn’s correction. Data are represented as mean ± SEM.

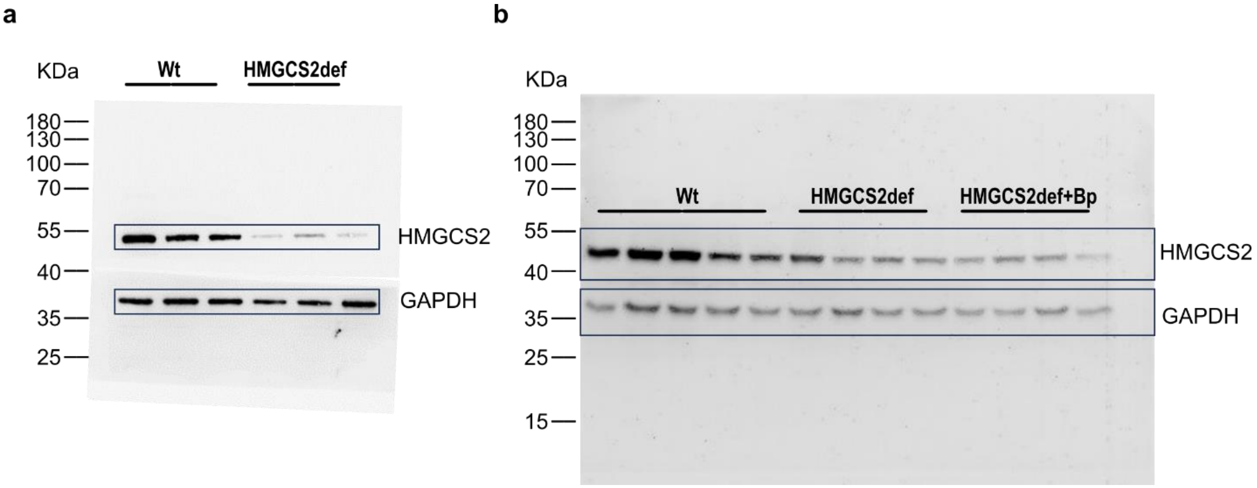

Supplementary Figure 11. HMGCS2 Expression in the liver.

(**a**) The expression of HMGCS2 proteins in the liver of control (Wt) and HMGCS2def mice was determined using Western blotting. (**b**) The HMGCS2 protein levels in liver of Wt, HMGCS2def and HMGCS2def+Bp mice at day 14 post-MI. The whole blot was separated into two parts for HMGCS2 (upper part) and GAPDH (lower part). HMGCS2 was detected with mouse monoclonal anti-HMGCS antibody (1:200; sc-393256; SANTA CRUZ, USA). GAPDH was detected with rabbit polyclonal anti-GAPDH antibodies (1:2000; cat no. 10494-1-AP; Proteintech, USA) and used as the loading control.

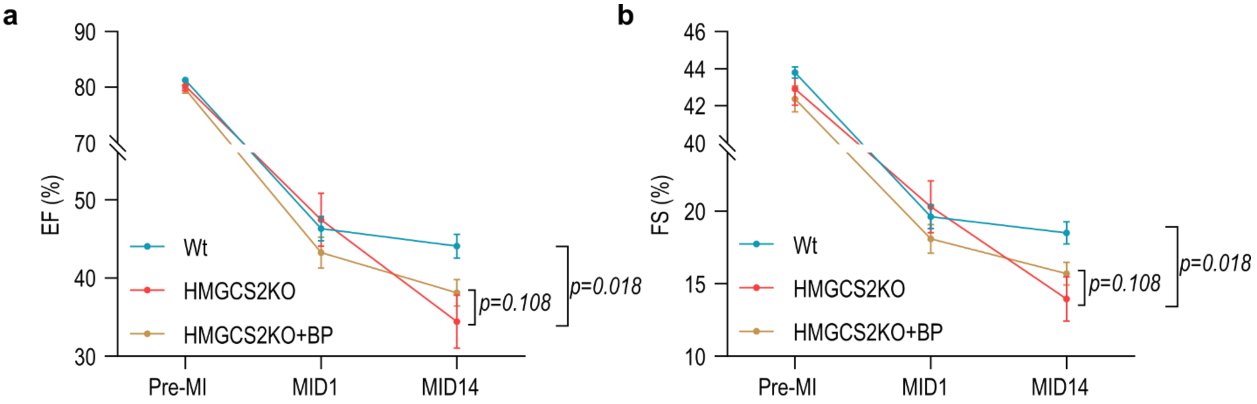

Supplementary Figure 12. Butyrate producers showed cardioprotective function in HMGCS2def mice.

(**a**) Echocardiographic analysis of the left ventricular EF (%) of HMGCS2def mice with/without butyrate producers (Bp) at pre-MI, post-MI day 1 (MID1) and post-MI day 14 (MID14). (**b**) Echocardiographic analysis of the left ventricular FS (%) of HMGCS2def mice at pre-MI, MID1 and MID14. Data were analyzed with the Mixed-effects analysis followed by Tukey correction and represented as mean ± SEM.
