# Supplementary material for: Gut butyrate-producers confer post-infarction cardiac protection
Source: Nat Commun. 2023 Nov 9;14:7249. doi: 10.1038/s41467-023-43167-5 (PMC10636175; doi:10.1038/s41467-023-43167-5)
Supplement: Supplementary file 3 — Description of Additional Supplementary Files [file 41467_2023_43167_MOESM3_ESM.docx]

Description of Additional Supplementary Files

**Gut butyrate-producers confer post-infarction cardiac protection**

Hung-Chih Chen *et al.*

Corresponding author: Patrick C.H. Hsieh, phsieh@ibms.sinica.edu.tw

File name: Supplementary Data 1

Description: The taxa data for supervised machine learning. These include the parameters used in the process, the reads at all taxa level and the reads at taxa level of genus.

File name: Supplementary Data 2

Description: The source codes for supervised machine learning. These include the code for jupyter notebook, python code and the code to set environment for anaconda.
