# Supplementary material for: Gut butyrate-producers confer post-infarction cardiac protection
Source: Nat Commun. 2023 Nov 9;14:7249. doi: 10.1038/s41467-023-43167-5 (PMC10636175; doi:10.1038/s41467-023-43167-5)
Supplement: Supplementary file 5 — Supplementary Data 2 [file 41467_2023_43167_MOESM5_ESM.docx]

**Source codes**

Code for jupyter notebook

{

"cells": [

{

"cell_type": "code",

"execution_count": null,

"id": "ebc2eddb-24b7-43e5-9a2a-f8bc8f211af4",

"metadata": {},

"outputs": [],

"source": [

"from pycaret.classification import *\n",

"import pandas as pd\n",

"para = pd.read_csv(\"parameters.csv\")\n",

"\n",

"for j in range(para.shape[0]):\n",

" for i in range(10):\n",

" outfilename = f\"result_{para['features'][j]}_{para['type'][j]}_{i}.csv\"\n",

" print(i,j,outfilename)\n",

" data = pd.read_csv(para['file'][j])\n",

" grid = setup(data=data, target='class', numeric_features=data.columns[1:].to_list(), session_id=i, \n",

" normalize = bool(para['normalize'][j]), normalize_method = para['method'][j], n_jobs = 10,\n",

" html=False, silent=True, verbose=False)\n",

" top3 = compare_models(verbose=False, n_select=3 )\n",

" tuned_top3 = [tune_model(k,choose_better=True) for k in top3]\n",

" cali_top3 = [calibrate_model(k) for k in tuned_top3]\n",

" blender = blend_models(cali_top3)\n",

" stacker = stack_models(cali_top3)\n",

" auto_model = automl()\n",

" create_model(auto_model, return_train_score=True)\n",

" df1 = pull()\n",

" create_model(auto_model, cross_validation = False)\n",

" df1.append(pull().rename(index={0:\"test\"})).to_csv(outfilename)"

]

},

{

"cell_type": "code",

"execution_count": null,

"id": "3dabab8a-f556-4cbd-8319-dadb2586a117",

"metadata": {},

"outputs": [],

"source": []

}

],

"metadata": {

"kernelspec": {

"display_name": "Python 3 (ipykernel)",

"language": "python",

"name": "python3"

},

"language_info": {

"codemirror_mode": {

"name": "ipython",

"version": 3

},

"file_extension": ".py",

"mimetype": "text/x-python",

"name": "python",

"nbconvert_exporter": "python",

"pygments_lexer": "ipython3",

"version": "3.8.13"

}

},

"nbformat": 4,

"nbformat_minor": 5

}

Python code

from pycaret.classification import *

import pandas as pd

para = pd.read_csv("parameters.csv")

for j in range(para.shape[0]):

for i in range(10):

outfilename = f"result_{para['features'][j]}_{para['type'][j]}_{i}.csv"

print(i,j,outfilename)

data = pd.read_csv(para['file'][j])

grid = setup(data=data, target='class', numeric_features=data.columns[1:].to_list(), session_id=i,

normalize = bool(para['normalize'][j]), normalize_method = para['method'][j], n_jobs = 20,

html=False, silent=True, verbose=False)

top3 = compare_models(verbose=False, n_select=3 )

tuned_top3 = [tune_model(k,choose_better=True) for k in top3]

cali_top3 = [calibrate_model(k) for k in tuned_top3]

blender = blend_models(cali_top3)

stacker = stack_models(cali_top3)

auto_model = automl()

create_model(auto_model, return_train_score=True)

df1 = pull()

create_model(auto_model, cross_validation = False)

df1.append(pull().rename(index={0:"test"})).to_csv(outfilename)

Environment setting for anaconda

name: pycaret_2_3_10

channels:

- defaults

dependencies:

- argon2-cffi=21.3.0=pyhd3eb1b0_0

- argon2-cffi-bindings=21.2.0=py38h2bbff1b_0

- babel=2.9.1=pyhd3eb1b0_0

- backcall=0.2.0=pyhd3eb1b0_0

- beautifulsoup4=4.11.1=py38haa95532_0

- brotlipy=0.7.0=py38h2bbff1b_1003

- ca-certificates=2022.07.19=haa95532_0

- certifi=2022.9.24=py38haa95532_0

- cffi=1.15.1=py38h2bbff1b_0

- colorama=0.4.5=py38haa95532_0

- decorator=5.1.1=pyhd3eb1b0_0

- defusedxml=0.7.1=pyhd3eb1b0_0

- entrypoints=0.4=py38haa95532_0

- idna=3.4=py38haa95532_0

- importlib_resources=5.2.0=pyhd3eb1b0_1

- ipython_genutils=0.2.0=pyhd3eb1b0_1

- jedi=0.18.1=py38haa95532_1

- json5=0.9.6=pyhd3eb1b0_0

- jsonschema=4.16.0=py38haa95532_0

- jupyter_client=7.3.5=py38haa95532_0

- jupyter_core=4.11.1=py38haa95532_0

- jupyter_server=1.18.1=py38haa95532_0

- jupyterlab=3.4.4=py38haa95532_0

- jupyterlab_pygments=0.1.2=py_0

- jupyterlab_server=2.15.2=py38haa95532_0

- libsodium=1.0.18=h62dcd97_0

- markupsafe=2.1.1=py38h2bbff1b_0

- matplotlib-inline=0.1.6=py38haa95532_0

- openssl=1.1.1q=h2bbff1b_0

- packaging=21.3=pyhd3eb1b0_0

- pandocfilters=1.5.0=pyhd3eb1b0_0

- parso=0.8.3=pyhd3eb1b0_0

- pickleshare=0.7.5=pyhd3eb1b0_1003

- pip=22.2.2=py38haa95532_0

- pkgutil-resolve-name=1.3.10=py38haa95532_0

- powershell_shortcut=0.0.1=3

- prometheus_client=0.14.1=py38haa95532_0

- pure_eval=0.2.2=pyhd3eb1b0_0

- pycparser=2.21=pyhd3eb1b0_0

- pyopenssl=22.0.0=pyhd3eb1b0_0

- pyparsing=3.0.9=py38haa95532_0

- pysocks=1.7.1=py38haa95532_0

- python=3.8.13=h6244533_0

- python-dateutil=2.8.2=pyhd3eb1b0_0

- python-fastjsonschema=2.16.2=py38haa95532_0

- requests=2.28.1=py38haa95532_0

- send2trash=1.8.0=pyhd3eb1b0_1

- six=1.16.0=pyhd3eb1b0_1

- soupsieve=2.3.2.post1=py38haa95532_0

- sqlite=3.39.3=h2bbff1b_0

- stack_data=0.2.0=pyhd3eb1b0_0

- testpath=0.6.0=py38haa95532_0

- tornado=6.2=py38h2bbff1b_0

- typing_extensions=4.3.0=py38haa95532_0

- urllib3=1.26.12=py38haa95532_0

- vc=14.2=h21ff451_1

- vs2015_runtime=14.27.29016=h5e58377_2

- wcwidth=0.2.5=pyhd3eb1b0_0

- wheel=0.37.1=pyhd3eb1b0_0

- win_inet_pton=1.1.0=py38haa95532_0

- wincertstore=0.2=py38haa95532_2

- winpty=0.4.3=4

- zeromq=4.3.4=hd77b12b_0

- pip:

- adagio==0.2.4

- aiohttp==3.8.3

- aiosignal==1.2.0

- alembic==1.8.1

- ansi2html==1.8.0

- antlr4-python3-runtime==4.11.1

- anyio==3.6.2

- appdirs==1.4.4

- asttokens==2.0.8

- async-timeout==4.0.2

- attrs==22.1.0

- autopage==0.5.1

- autoviz==0.1.58

- azure-core==1.26.0

- azure-storage-blob==12.14.1

- bcrypt==4.0.1

- bleach==5.0.1

- blis==0.7.9

- bokeh==2.4.3

- boruta==0.3

- boto3==1.24.96

- botocore==1.27.96

- brotli==1.0.9

- cachetools==5.2.0

- catalogue==1.0.2

- catboost==1.1

- charset-normalizer==2.1.1

- chart-studio==1.1.0

- click==8.0.4

- cliff==4.0.0

- cloudpickle==2.2.0

- cmaes==0.8.2

- cmd2==2.4.2

- colorcet==3.0.1

- colorlog==6.7.0

- colorlover==0.3.0

- colour==0.1.5

- cryptography==38.0.1

- cufflinks==0.17.3

- cycler==0.11.0

- cymem==2.0.7

- cython==0.29.14

- dash==2.6.2

- dash-auth==1.4.1

- dash-bootstrap-components==0.13.1

- dash-core-components==2.0.0

- dash-cytoscape==0.3.0

- dash-html-components==2.0.0

- dash-table==5.0.0

- databricks-cli==0.17.3

- dataclasses==0.6

- debugpy==1.6.3

- dill==0.3.6

- distlib==0.3.6

- docker==6.0.0

- dtreeviz==1.4.0

- emoji==2.1.0

- evidently==0.1.51.dev0

- executing==1.1.1

- explainerdashboard==0.3.8.2

- fairlearn==0.7.0

- fastapi==0.85.1

- ffmpy==0.3.0

- filelock==3.8.0

- flask==2.1.3

- flask-compress==1.13

- flask-seasurf==1.1.1

- flask-simplelogin==0.1.1

- flask-wtf==0.15.1

- fonttools==4.38.0

- frozenlist==1.3.1

- fs==2.4.16

- fsspec==2022.10.0

- fugue==0.7.3

- fugue-sql-antlr==0.1.1

- funcy==1.17

- future==0.18.2

- gensim==3.8.3

- gevent==22.10.1

- gitdb==4.0.9

- gitpython==3.1.29

- google-api-core==2.10.2

- google-auth==2.13.0

- google-cloud-core==2.3.2

- google-cloud-storage==2.5.0

- google-crc32c==1.5.0

- google-resumable-media==2.4.0

- googleapis-common-protos==1.56.4

- gradio==3.6

- greenlet==1.1.3.post0

- grpcio==1.43.0

- h11==0.12.0

- holoviews==1.14.9

- htmlmin==0.1.12

- httpcore==0.15.0

- httpx==0.23.0

- hvplot==0.8.1

- hyperopt==0.2.7

- imagehash==4.3.1

- imageio==2.22.2

- imbalanced-learn==0.7.0

- importlib-metadata==5.0.0

- importlib-resources==5.10.0

- iniconfig==1.1.1

- interpret==0.2.4

- interpret-core==0.2.7

- ipykernel==6.16.1

- ipython==8.5.0

- ipywidgets==8.0.2

- isodate==0.6.1

- itsdangerous==2.1.2

- jinja2==3.1.2

- jmespath==1.0.1

- joblib==1.2.0

- jupyter==1.0.0

- jupyter-client==7.4.3

- jupyter-console==6.4.4

- jupyter-core==4.11.2

- jupyter-dash==0.4.2

- jupyter-server==1.21.0

- jupyterlab-pygments==0.2.2

- jupyterlab-widgets==3.0.3

- kiwisolver==1.4.4

- kmodes==0.12.2

- lightgbm==3.3.3

- lime==0.2.0.1

- linkify-it-py==1.0.3

- llvmlite==0.37.0

- m2cgen==0.10.0

- mako==1.2.3

- markdown==3.4.1

- markdown-it-py==2.1.0

- matplotlib==3.5.3

- mdit-py-plugins==0.3.1

- mdurl==0.1.2

- missingno==0.5.1

- mistune==2.0.4

- mlflow==1.30.0

- mlxtend==0.19.0

- msgpack==1.0.4

- msrest==0.7.1

- multidict==6.0.2

- multimethod==1.9

- multiprocess==0.70.14

- murmurhash==1.0.9

- nbclassic==0.4.5

- nbclient==0.7.0

- nbconvert==7.2.2

- nbformat==5.7.0

- nest-asyncio==1.5.6

- networkx==2.8.7

- nltk==3.7

- notebook==6.5.1

- notebook-shim==0.2.0

- numba==0.54.1

- numexpr==2.8.3

- numpy==1.20.3

- oauthlib==3.2.2

- optuna==2.10.1

- orjson==3.8.0

- oyaml==1.0

- pandas==1.5.1

- pandas-profiling==3.4.0

- panel==0.12.7

- param==1.12.2

- paramiko==2.11.0

- pathos==0.3.0

- patsy==0.5.3

- pbr==5.11.0

- phik==0.12.2

- pillow==9.2.0

- plac==1.1.3

- platformdirs==2.5.2

- plotly==5.10.0

- pluggy==1.0.0

- pox==0.3.2

- ppft==1.7.6.6

- preshed==3.0.8

- prettytable==3.4.1

- prometheus-client==0.15.0

- prometheus-flask-exporter==0.20.3

- prompt-toolkit==3.0.31

- protobuf==3.19.6

- psutil==5.9.3

- py==1.11.0

- py4j==0.10.9.7

- pyamg==4.2.3

- pyaml==21.10.1

- pyarrow==9.0.0

- pyasn1==0.4.8

- pyasn1-modules==0.2.8

- pycaret==2.3.10

- pycryptodome==3.15.0

- pyct==0.4.8

- pydantic==1.10.2

- pydub==0.25.1

- pygments==2.13.0

- pyjwt==2.6.0

- pyldavis==3.3.1

- pynacl==1.5.0

- pynndescent==0.5.7

- pyod==1.0.6

- pyperclip==1.8.2

- pyreadline3==3.4.1

- pyrsistent==0.18.1

- pytest==7.1.3

- python-graphviz==0.20.1

- python-multipart==0.0.5

- pytz==2022.5

- pyviz-comms==2.2.1

- pywavelets==1.4.1

- pywin32==304

- pywinpty==2.0.8

- pyyaml==5.4.1

- pyzmq==24.0.1

- qpd==0.3.3

- qtconsole==5.3.2

- qtpy==2.2.1

- querystring-parser==1.2.4

- ray==2.0.1

- regex==2022.9.13

- requests-oauthlib==1.3.1

- retrying==1.3.3

- rfc3986==1.5.0

- rsa==4.9

- s3transfer==0.6.0

- salib==1.4.5

- scikit-image==0.19.3

- scikit-learn==0.23.2

- scikit-optimize==0.9.0

- scikit-plot==0.3.7

- scipy==1.5.4

- seaborn==0.12.1

- setuptools==60.10.0

- shap==0.41.0

- sklearn==0.0

- skope-rules==1.0.1

- slicer==0.0.7

- smart-open==6.2.0

- smmap==5.0.0

- sniffio==1.3.0

- spacy==2.3.8

- sqlalchemy==1.4.42

- sqlparse==0.4.3

- srsly==1.0.6

- stack-data==0.5.1

- starlette==0.20.4

- statsmodels==0.13.2

- stevedore==4.1.0

- tabulate==0.9.0

- tangled-up-in-unicode==0.2.0

- tenacity==8.1.0

- tensorboardx==2.5.1

- terminado==0.16.0

- textblob==0.17.1

- thinc==7.4.6

- threadpoolctl==3.1.0

- tifffile==2022.10.10

- tinycss2==1.2.1

- tomli==2.0.1

- tqdm==4.64.1

- traitlets==5.5.0

- treeinterpreter==0.2.3

- triad==0.7.0

- tune-sklearn==0.4.4

- typing-extensions==4.4.0

- ua-parser==0.16.1

- uc-micro-py==1.0.1

- umap-learn==0.5.3

- uvicorn==0.19.0

- virtualenv==20.16.5

- visions==0.7.5

- waitress==2.1.2

- wasabi==0.10.1

- webencodings==0.5.1

- websocket-client==1.4.1

- websockets==10.3

- werkzeug==2.0.3

- widgetsnbextension==4.0.3

- wordcloud==1.8.2.2

- wtforms==3.0.1

- xgboost==1.6.2

- xlrd==2.0.1

- yarl==1.8.1

- yellowbrick==1.2.1

- zipp==3.10.0

- zope-event==4.5.0

- zope-interface==5.5.0

prefix: C:\ProgramData\Anaconda3\envs\pycaret_2_3_10
