# Supplementary material for: Gut butyrate-producers confer post-infarction cardiac protection
Source: Nat Commun. 2023 Nov 9;14:7249. doi: 10.1038/s41467-023-43167-5 (PMC10636175; doi:10.1038/s41467-023-43167-5)
Supplement: Supplementary file 6 — Reporting Summary [file 41467_2023_43167_MOESM6_ESM.pdf]

nature portfolio

Corresponding author(s): Patrick C.H. Hsieh

Last updated by author(s): Oct 20, 2023

Reporting Summary

Nature Portfolio wishes to improve the reproducibility of the work that we publish. This form provides structure for consistency and transparency in reporting. For further information on Nature Portfolio policies, see our Editorial Policies and the Editorial Policy Checklist.

Statistics

For all statistical analyses, confirm that the following items are present in the figure legend, table legend, main text, or Methods section.

n/a	Confirmed	
[ ]	[x]	The exact sample size (*n*) for each experimental group/condition, given as a discrete number and unit of measurement
[ ]	[x]	A statement on whether measurements were taken from distinct samples or whether the same sample was measured repeatedly
[ ]	[x]	The statistical test(s) used AND whether they are one- or two-sided<br>*Only common tests should be described solely by name; describe more complex techniques in the Methods section.*
[ ]	[x]	A description of all covariates tested
[ ]	[x]	A description of any assumptions or corrections, such as tests of normality and adjustment for multiple comparisons
[ ]	[x]	A full description of the statistical parameters including central tendency (e.g. means) or other basic estimates (e.g. regression coefficient) AND variation (e.g. standard deviation) or associated estimates of uncertainty (e.g. confidence intervals)
[ ]	[x]	For null hypothesis testing, the test statistic (e.g. *F*, *t*, *r*) with confidence intervals, effect sizes, degrees of freedom and *P* value noted<br>*Give P values as exact values whenever suitable.*
[x]	[ ]	For Bayesian analysis, information on the choice of priors and Markov chain Monte Carlo settings
[x]	[ ]	For hierarchical and complex designs, identification of the appropriate level for tests and full reporting of outcomes
[x]	[ ]	Estimates of effect sizes (e.g. Cohen's *d*, Pearson's *r*), indicating how they were calculated

Our web collection on statistics for biologists contains articles on many of the points above.

Software and code

Policy information about availability of computer code

Data collection	Pannoramic scanner software, Topspin 2.1
Data analysis	MicrobiomeAnalyst, QIIME 2 (version 2020.11), Silva database v.138, Galaxy, Bowtie2 (v2.3.5.1), MEGAHIT (v1.1.3), CD-HIT (v4.7), BWA (v0.7.17-r1188), DIAMOND software (0.9.22), KEGG, GhostKOALA, MetaboAnalyst5.0, Chenomx NMR suite 8.5, Waters Empower 3, ImageJ, PyCaret package, GraphPad Prism 8

For manuscripts utilizing custom algorithms or software that are central to the research but not yet described in published literature, software must be made available to editors and reviewers. We strongly encourage code deposition in a community repository (e.g. GitHub). See the Nature Portfolio guidelines for submitting code & software for further information.

Data

Policy information about availability of data

All manuscripts must include a data availability statement. This statement should provide the following information, where applicable:

- Accession codes, unique identifiers, or web links for publicly available datasets
- A description of any restrictions on data availability
- For clinical datasets or third party data, please ensure that the statement adheres to our policy

The raw data generated in this study are provided in the Source Data file. The LC-MS data used in this study are available in the MetaboLights database under accession code MTBLS8802 [www.ebi.ac.uk/metabolights/MTBLS8802]. The raw sequences used in this study are available in the NCBI SRA database [Accession number: PRJNA1031545]. The code for machine learning is available in the Zenodo database [DOI: DOI: 10.5281/zenodo.10032793].

Human research participants

Policy information about studies involving human research participants and Sex and Gender in Research.

Reporting on sex and gender	The recruitment of control and STEMI patients was on availability; both sexes of control and STEMI patients were recruited.
Population characteristics	Please see Extended Data Table 1 for the clinical characteristics of the STEMI patients recruited.
Recruitment	STEMI patients and controls were recruited in the hospital when candidates fulfilled the recruitment criteria and signed the informed consent forms. The STEMI status was diagnosed with electrocardiography and catheterization by board-certified cardiologists at three medical centers in Taiwan before recruitment. The selection bias is that it includes only patients referred for electrocardiography and PCI. This selection criteria exclude patients with impending STEMI, leading to small cohort size. However, patients referred for electrocardiography and PCI have confirmed STEMI which make the study cohort more homogeneous.
Ethics oversight	Institutional Review Board, National Cheng-Kung University Hospital (NCKUH; 8800-4-03-005), Taiwan; Research Ethics Review Committee, China Medical University & Hospital (CMUH; fCMUH108-REC3-016(CR-2)), Taiwan; Research Ethics Review Committee, Far Eastern Memorial Hospital (FEMH; FEMH, 107175-E), Taiwan; IRB on Biomedical Science Research, Academia Sinica (AS-IRB02-110151), Taiwan.

Note that full information on the approval of the study protocol must also be provided in the manuscript.

Field-specific reporting

Please select the one below that is the best fit for your research. If you are not sure, read the appropriate sections before making your selection.

☒ Life sciences ☐ Behavioural & social sciences ☐ Ecological, evolutionary & environmental sciences

For a reference copy of the document with all sections, see nature.com/documents/nr-reporting-summary-flat.pdf

Life sciences study design

All studies must disclose on these points even when the disclosure is negative.

Sample size	The sample size was determined based on previous studies within the lab using these techniques with alpha equals to 0.05, beta equals to 0.2. see the references PMID: 30586712, PMID: 36420731 and PMID: 37732466.
Data exclusions	No data were systematically excluded.
Replication	The reported findings were replicated across multiple biological samples (n was indicated in the figures). Experimental findings were reliably reproduced.
Randomization	Randomization was not relevant in the human study because the investigators were comparing well-defined STEMI patients with controls and the human control and STEMI patients were recruited on availability. For mice and nonhuman primate experiments, animals were randomized.
Blinding	Investigators were blinded for experiments and analysis, including histological analysis

Reporting for specific materials, systems and methods

We require information from authors about some types of materials, experimental systems and methods used in many studies. Here, indicate whether each material, system or method listed is relevant to your study. If you are not sure if a list item applies to your research, read the appropriate section before selecting a response.

Materials & experimental systems

n/a	Involved in the study
[ ]	[x] Antibodies
[x]	[ ] Eukaryotic cell lines
[x]	[ ] Palaeontology and archaeology
[ ]	[x] Animals and other organisms
[x]	[ ] Clinical data
[x]	[ ] Dual use research of concern

Methods

n/a	Involved in the study
[x]	[ ] ChIP-seq
[x]	[ ] Flow cytometry
[x]	[ ] MRI-based neuroimaging

Antibodies

Antibodies used

mouse monoclonal mHMGCS Antibody (B-8) (1:200; Cat No: sc-393256; Santa Cruz), Rabbit anti-GAPDH Polyclonal antibody (1:2,000; Cat No: 10494-1-AP; Proteintech Group, Inc); Goat Anti-Mouse IgG antibody (HRP)(1:1,000; Cat No: GTX213111-01; GeneTex), HRP-conjugated IgG Fraction Monoclonal Mouse Anti-Rabbit IgG (Light Chain Specific)(1:4,000; Cat No:SA00001-7L; Proteintech Group, Inc)

Validation

mouse monoclonal mHMGCS Antibody: see specification statement from the manufacturer website https://www.scbt.com/p/mhmgcs-antibody-b-8
Rabbit anti-GAPDH Polyclonal antibody: see specification statement from manufacturer website https://www.ptglab.com/products/GAPDH-Antibody-10494-1-AP.htm
Goat Anti-Mouse IgG antibody (HRP): see specificity statement from manufacturer website https://www.genetex.com/Product/Detail/Goat-Anti-Mouse-IgG-antibody-HRP/GTX213111-01
HRP-conjugate IgG Fraction Monoclonal anti-Rabbit IgG: see specificity statement from manufacturer website https://www.ptglab.com/products/HRP-Mouse-Anti-Rabbit-IgG-Light-Chain-Specific-secondary-antibody.htm

Animals and other research organisms

Policy information about studies involving animals; ARRIVE guidelines recommended for reporting animal research, and Sex and Gender in Research

Laboratory animals

10-12 week-old C57Bl/6J specific pathogen free (SPF) and germ free (GF) mice, 10-year old Macaque rhesus

Wild animals

This study did not involve wild animals.

Reporting on sex

Only male mice and Macaque were used in this study as the high level of estrogen in female animals may interfere the interpretation of cardiac protection function of any treatment.

Field-collected samples

this study did not involve field-collected samples.

Ethics oversight

The mouse experiments in this study were approved by Academia Sinica Institutional Animal Care and Use Committee and NLAC Animal Care and Use Committee respectively (IACUC No. 18041211). The nonhuman primate experiment was approved by the experimental
animal committee of UW-Madison (IACUC No. G006084-A07).

Note that full information on the approval of the study protocol must also be provided in the manuscript.
